# Soccer Offside Judgments in Laypersons with Different Types of Static Displays

**DOI:** 10.1371/journal.pone.0133687

**Published:** 2015-08-07

**Authors:** Peter Wühr, Frowin Fasold, Daniel Memmert

**Affiliations:** 1 Institute of Psychology, TU Dortmund University, Dortmund, Germany; 2 Institute of Cognitive and Team/Racket Sport Research, German Sport University, Cologne, Germany; University of Akron, UNITED STATES

## Abstract

Four experiments investigated offside decisions in laypersons with different types of static displays. Previous research neglected this group although the majority of assistant referees in soccer games at the amateur level are laypersons. The aims of our research were (a) to investigate the spatial resolution in laypersons’ perception of offside situations, (b) to search for biases in laypersons’ offside judgments, and (c) to develop useful displays for future research. The displays showed the moment when a midfielder passes the ball to a forward moving in the vicinity of a defender. We varied the spatial location of the forward around the defender in eleven steps and participants made their offside decision by pressing a key. Across experiments, displays varied in abstractness (simple shapes, clipart figures, photographs). There were two major findings. Firstly, both accuracy and speed of offside judgments deteriorated when the spatial distance between forward and defender decreased, approaching guessing rate at the smallest distances. Secondly, participants showed a consistent bias in favor of the non-offside response, in contrast to most studies on professional assistant referees. In sum, the results highlight the limited spatial resolution of the visual system and underscore the role of response bias in offside-judgment tasks.

## Introduction

On every weekend, millions of football (or soccer) fans around the world discuss the performance of players and referees, and especially referees are often criticized for wrong decisions. However, refereeing a soccer match is an extremely difficult task. The basic requirements involve a high degree of physical fitness, and extensive knowledge of the rules of the game. Moreover, applying the rules to the game in an appropriate manner is a cognitively highly demanding task. In particular, referees must anticipate, perceive and evaluate complex and dynamic game situations, and communicate their decision within a few seconds (for a review, see [1]). The task is further complicated by the fact that, during a soccer match, several critical events may occur at the same time at different places, requiring referees to divide attention. This is one reason for why, at the professional level, the main referee is supported by two assistant referees, one on each side of the soccer field. In addition, the presence and behavior of trainers, substitute players and the crowd may also interfere with the referees’ task.

A major duty of the assistant referees is to support the main referee in applying the “offside” rule, as defined by *FIFA* (Fédération Internationale de Football Association). The offside rule says, “A player is in an offside position if he is nearer to his opponents’ goal line than both the ball and the second-last opponent. […] A player in an offside position is only penalized if, at the moment the ball touches or is played by one of his team, he is, in the opinion of the referee, involved in active play by interfering with play or interfering with an opponent or gaining an advantage by being in that position.”[2]. The term “second-last opponent” includes every player of the defending team. The major purpose of the offside rule is to force the attacking players to start their attack (i.e. attempt to score a goal) before or at a line defined by the position of the second-last opponent in the field. Offside (or the offside line) is not defined with regard to the last opponent because, in most cases, the last opponent is the goal keeper that is usually standing on the goal line. Hence, if the offside rule was defined with regard to the last opponent, there would be no offside in most of the cases (where the goal keeper or the last defender is standing on his/her goal line).

The phrase “nearer to his opponents’ goal line” refers to those body parts that could be used to score a goal (i.e. all body parts except arms and hands). It follows from the rule that judging offside includes two subtasks. The first subtask requires perceiving the position of the forward from team A in relation to the last defender of team B (who is, in most cases, the second last opponent before the goal keeper) at the moment when another player from team A touches or plays the ball. The second subtask requires judging whether the forward is actively involved in play or gaining an advantage from being in the offside position.

During the last decade, empirical research on offside judgments mainly addressed the first subtask of perceiving the position of a forward in relation to the last defender when the ball is played. In most studies, researchers analyzed film or video material of professional soccer matches from national leagues or from international tournaments in order to determine (a) the accuracy of offside judgments by assistant referees, and (b) variables that are responsible for errors in these judgments. This research revealed that perceptual illusions [3], the speed and position of the assistant referee [3, 4], and gaze shifts of the assistant referee across large distances [5, 6] may lead to errors in offside judgments. Notably, there is almost no empirical research on offside judgments in laypersons, although laymen often serve as ad-hoc assistant referees in the great majority of soccer matches at the amateur level. For example, in Germany, there are only 27 official matches in three professional soccer leagues per week, in contrast to several thousand soccer matches in over 2,000 amateur divisions per week.

From a psychological viewpoint, the task of judging offside is a perceptual categorization task that requires people to sort an infinite set of game situations into one of two possible categories (non-offside situations vs. offside situations). The factorial combination of two possible situations (forward is not offside; forward is offside) and two possible judgments (not offside; offside) reveals two types of correct decisions and two types of errors in judging offside, respectively (cf. Table 1). The first error occurs when the assistant referee judges a player to be offside who is, in fact, not offside. This error is called a “false alarm” or “flag error” because the assistant referee typically signals offside judgments by lifting a flag. The second error occurs when the assistant referee judges a player not to be offside who is, in fact, offside. This error is called a “miss” or “non-flag error”.

**Table 1:** The table lists the four possible outcomes of an offside decision in terms of Signal-Detection Theory [19]. The terms in brackets (flag error vs. non-flag error) are commonly used in publications on offside judgments.

		Judgment	
		No Offside	Offside
**Situation**	**No Offside**	Correct Rejection	False Alarm (Flag Error)
	**Offside**	Miss (Non-Flag Error)	Hit

Research showed that even expert (assistant) referees exhibit considerable error rates in offside judgments that vary around 20%. For example, one study reported an overall error rate of 26.2% for FIFA assistant referees when judging offside during the 64 matches played during 2002 FIFA world cup [3]. Similarly, a further investigation observed an overall error rate of 17.5% when analyzing performance of assistant referees in 165 matches from the English Premier League during the 2007–2008 [7] season. The majority of studies observed that flag errors were more frequent than non-flag errors [4, 8–11]. However, in the FIFA world cup 2002 similar numbers of flag and non-flag errors were observed [3], and even in matches of the Premier League more non-flag errors than flag-errors were reported [7].

Four accounts–that are not mutually exclusive–have been proposed to explain the occurrence, and distribution, of errors in offside judgments. The *gaze-shift hypothesis* attributes errors to the time required for shifting the referees’ gaze from the player touching the ball to the teammate that is nearest to the opponents’ goal line [5, 6]. Hence, if the forward moves from a non-offside position to an offside position during the gaze shift, the assistant referee will miss the movement because of saccadic suppression and judge offside with regard to the forwards’ position after the shift. Hence, the gaze-shift hypothesis predicts a preponderance of flag errors in offside judgments. However, research with eye-tracking methodology revealed that assistant referees do not fixate the ball in offensive situations, but fixate a point close to the offside line instead [12].

The *flash-lag hypothesis* attributes errors in offside judgments to misperceptions in the location of moving stimuli [13]. In a typical laboratory task, participants have to indicate the location of a moving stimulus at a point in time as defined by a short, local flash. The typical result is a mislocalization of the moving stimulus in the direction of its movement–an observation called the ‘flash-lag effect’ [14, 15]. The flash-lag effect may explain errors in judging offside because it suggests that, when the ball is played, the location of a moving forward is misperceived as being closer to the goal line than he actually is [13]. The problem should be particularly eminent when the defender stands still, or even moves in the opposite direction [10]. The flash-lag hypothesis predicts that flag errors should be more frequent than non-flag errors and provides a possible explanation for flag errors, but it cannot account for non-flag errors.

The *optical-error hypothesis* relates errors in offside judgments to the configuration of the forward, the second-last defender, and the assistant referee with regard to the offside line [16, 17]. The “offside line” is an imaginary line through the second-last defender that separates the field into an offside region (between the second-last defender and the goal of his/her team) and a non-offside region (between the second-last defender and his/her opponents’ goal). This hypothesis rests on the fact that assistant referees are often positioned ahead of or behind the offside line, which has implications for their judgments. In particular, when the assistant referee is positioned ahead of the offside line and the forward is farther away from him than the defender, the forward may erroneously be perceived to be offside because he appears closer to the goal than the defender, increasing the likelihood of a flag error. When the assistant referee is positioned ahead of the offside line and the forward is closer to him than the defender, the forward may erroneously be perceived non-offside because he appears farther from the goal than the defender, increasing the likelihood of a non-flag error. The reverse is true when the assistant referee is positioned behind the offside line. Moreover, according to the first investigations of this account [16], optical errors are more likely when the forward is farther away from the assistant referee than when the forward is closer to the assistant referee. Therefore, flag errors should be more likely when the assistant referee is positioned ahead of the offside line, whereas non-flag errors should be more likely when the assistant referee is positioned behind the offside line. Since the first publication [16], empirical evidence has been presented in favor of the optical-error hypothesis [3, 17, 18].

A fourth account for the occurrence and distribution of errors in offside judgments arises from *signal-detection theory* (SDT) [19, 20]. SDT provides a theoretical framework and mathematical methods for analyzing decisions about ambiguous inputs in perception. According to SDT, two major variables affect any sort of perceptual categorization: sensitivity and response bias. *Sensitivity* is the ability to discriminate between the members of different categories. This ability is expressed by means of the sensitivity index *d’* (called: d prime). *Response bias* is the preference for a particular category (or response). The response bias is expressed in the bias index *c*. According to SDT, errors in judging offside should arise if a category is preferred (bias) or when the perceptual sensitivity for discriminating between offside and non-offside situations is low. Please note that, besides *d'* and *c*, other measures for perceptual sensitivity and response bias are available. Several measures and their parametric properties are critical discussed in [38].

The indices *d’* and *c* can be estimated from empirical data. To understand this estimation, we must consider the four possible outcomes when judging potential offside situations (cf. Table 1). If an assistant referee wants to detect as many offside situations as possible, he will prefer the offside response over the non-offside response in doubtful situations. This strategy will increase the “hit” rate (i.e. the number of correctly identified offside situations), but also the false-alarm rate, and results in more flag than non-flag errors. In contrast, if an assistant referee wants to avoid false-alarms (i.e. flag errors), he will prefer the non-offside response over the offside response in doubtful situations. This strategy will increase the correct-rejection rate (i.e. the number of correctly identified non-offside situations), but also the miss rate, and results in more non-flag errors than flag errors.

The sensitivity index *d’* demonstrates the difference between the (normalized) hit rate and the (normalized) false-alarm rate. The index *d’* represents the participants’ ability to perceptually discriminate offside from non-offside situations. Discrimination accuracy (and *d’*) is zero if the hit rate equals the false-alarm rate. Perfect discrimination, on the other hand, implies an infinite *d’*. When hit and false-alarm rates have two decimal places, however, the largest finite value of *d’* is 4.65, and this can be viewed as an effective ceiling (cf. [20]). The response-bias *c* represents the ratio of the two responses in two-alternative forced choice tasks, with a value of 0 representing the absence of a bias towards one or the other response. By means of this bias SDT can explain differences between the relative frequency of flag errors (“false alarms”) and non-flag errors (“misses”) [1, 21].

A response bias may have different sources that could be classified into extrinsic and intrinsic sources. *Extrinsic* sources are features of a particular game (or task) that could bias an observer (or assistant referee) to prefer one response over the other. Extrinsic sources include different base rates for offside and non-offside situations or different consequences for flag and non-flag errors. Different base rates are present when, for example, an experiment (or soccer match) contains 75% non-offside situations and only 25% offside situations rather than 50% of each situation [10]. Observers might recognize the difference in base rates and accordingly match the proportion of offside to non-offside responses in doubtful situations. The to-be-expected result would be a preference for the non-offside response and, therefore, a preponderance of non-flag errors in doubtful situations. The fact that the results from an off-the-field test [10] showed a preponderance of flag errors indicates that their participants did not recognize the different base rates and/or had a stronger preference for the offside response.

*Intrinsic* sources are features of a particular observer (or assistant referee) that make him or her prefer one response over the other. Intrinsic sources include the observers’ attitude towards the game or learned instructions for how to decide in perceptually unclear situations. For example, the observer might hold the attitude that, when in doubt, he/she should decide in favor of keeping the game running and thus does not interrupt. Assistant referees receive no written law, only a norm from the federations, to decide in favor of the forward when in doubt [7]. This makes the game more attractive. Similarly, the instruction (or wish) to interrupt a soccer match as rarely as possible could produce a bias against the offside response (and a preponderance of non-flag errors, as observed in the English Premier League [7]).

To summarize, there is a limited number of empirical studies on the cognitive determinants of offside judgments in soccer. Typically, these studies investigated the performance of expert (assistant) referees, and most of these studies involved the analysis of film or video recordings from real soccer games. Only recently, researchers started investigating offside judgments in laboratory experiments [10, 11, 12]. Methods used in these experimental studies involved the presentation of original film or video material [10], or the presentation of computer-animated game situations to the participants [10, 11]. Together, these studies already identified some critical factors influencing offside decisions (e.g., relative positions of forward, defender, and assistant referee, with regard to each other and the offside line; movement direction of defender; speed of assistant referee), indicating the complexity of both the task of judging offside and the challenge of investigating its cognitive determinants. Moreover, there are already some interesting accounts of errors in offside decisions, but these accounts are not yet sufficient to explain the complete pattern of available data. We suggest using SDT in research on offside decisions because it represents a very powerful conceptual framework for guiding this research, and provides useful methods for performing it [1, 21].

The present study had two major aims that were different from those of previous studies. The first aim of our study was to investigate offside judgments in *laypersons*, and not in experts. This was done for two reasons. Firstly, previous studies exclusively investigated performance of expert referees, but expert referees and professional assistant referees are only involved in a small subset of (professional) soccer games in everyday life. In contrast, the vast majority of soccer matches in amateur soccer leagues is led by amateur referees, who are often assisted by ad hoc referees, who do not have much expertise in refereeing matches and judging offside situations. Secondly, although there certainly are effects of expertise on offside judgments [10], some basic perceptual and cognitive processes serving this task are presumably similar in experts and novices (e.g. [22, 23, 24]). Hence, it should be possible to identify basic characteristics of offside judgments from studying performance of laypersons. Whether these characteristics also occur in expert behavior is an empirical question for subsequent research.

We were particularly interested in three aspects of laypersons’ offside judgments. The first aspect concerned the *spatial resolution* in judging offside situations. The ability to perceptually discriminate offside from non-offside situations should certainly depend on the spatial distance between the forward and the second last defender who defines the offside line. Interestingly, little empirical work has been devoted to this important variable. Only one study examined this factor in four steps [10]. In computer animations, the forward was either 20 pixels behind the offside line, 10 pixels behind the offside line, at the offside line, or 10 pixels ahead of the offside line, and this variable was crossed with two additional variables, namely, the action of the defender (static or moving), and the speed of movement (6 vs. 8 frames per second). Interestingly, these authors found the majority of errors when the forward was close behind (24%) or at the offside line (60%), compared to the conditions with the forward far behind the offside line (10%) or ahead of the offside line (11%). Two aspects of these results are noteworthy: The observed error rate at the offside line underscores the guessing rate (50%) and errors are not symmetrically distributed around the offside line. This pattern is consistent with the flash-lag hypothesis, but a response bias might have contributed as well. In our experiments, we manipulated the spatial distance between the defender and the forward in 11 steps that were centered on the offside line.

The second interesting aspect of laypersons offside judgments concerned the presence of a *response bias* in this population. The presence of a response bias in our participants would underscore the importance of this variable in offside research. Therefore, in each experiment, we analyzed the performance of our participants according to SDT, and computed both the sensitivity index *d’* and the bias index *c*. Note that, in our task, the presence of a response bias would lead to an asymmetric distribution of errors around the condition with minimal spatial separation between forward and defender.

The third interesting aspect of laypersons’ offside judgments concerned the effectiveness of immediate response *feedback*. The possible effect of feedback was addressed in Experiment 3. A couple of previous studies observed positive effects of feedback on referee decisions, but these studies typically involved several training sessions with feedback. For example, two studies showed that presenting the critical game situation again immediately after the assistant referee had made his decision improved offside judgments [25, 26]. Another study investigated the impact of simple feedback (correct vs. incorrect) on the accuracy of foul decisions over a three-week training period [27]. Results showed that training improved performance from a pre-test to a post-test in both novices and expert referees. Hence, previous research demonstrated that providing feedback can improve referee decisions, but the effect of immediate response feedback was actually not investigated. Therefore, we explored whether and how immediate response feedback would affect perceptual sensitivity and/or the response bias in offside judgments. Providing feedback might either increase sensitivity or reduce a response bias (or both).

**Fig 1:**
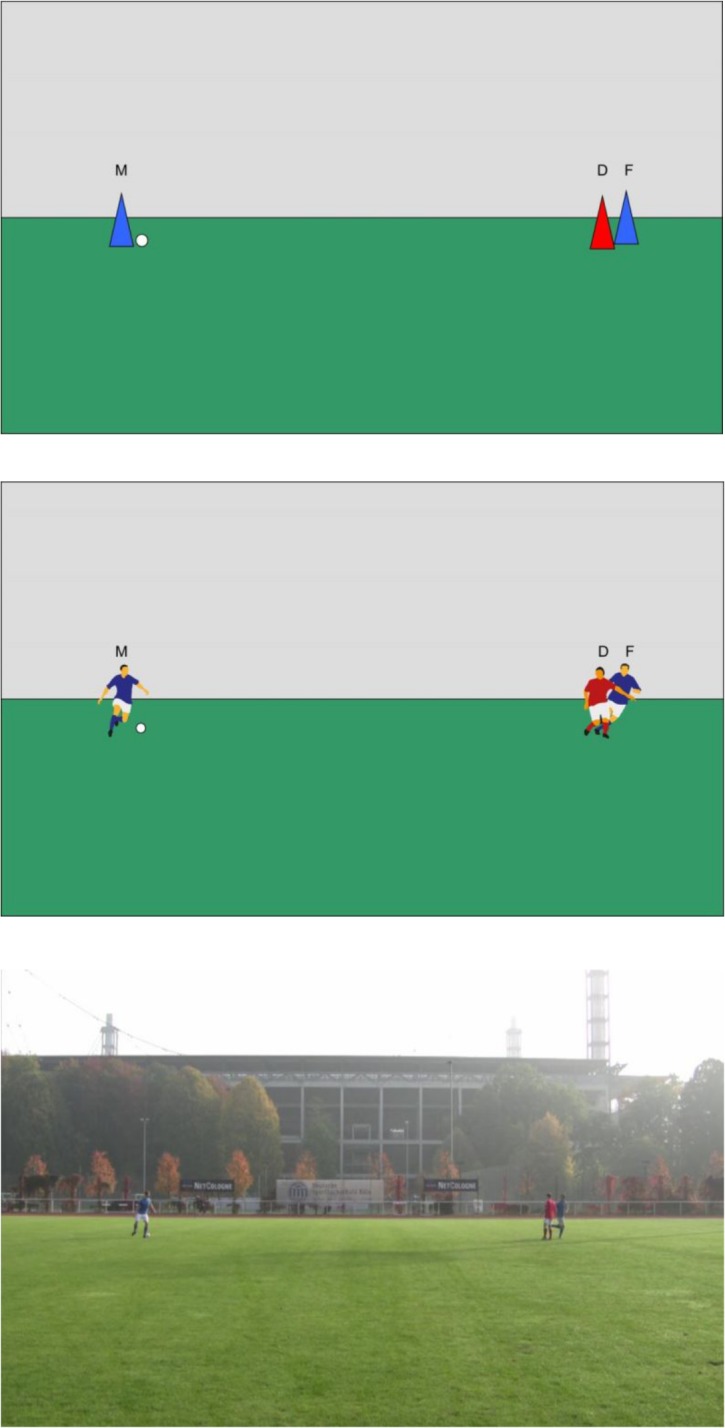
Sample displays from our experiments. Each display shows a situation where the blue team playing from left to right, and the blue forward is standing offside because he is closer to the red teams’ goal line than the red defender. For matters of illustration, the midfielder is labelled “M”, the defender is labelled “D” and the forward is labelled “F” in the sample displays, but these labels were *not* presented to participants. The Displays of the type shown in Fig 1A were used in Experiment 1A/B. Displays of the type shown in Fig 1B were used in Experiments 2 and 3. Displays of the type shown in Fig 1C were used in Experiment 4.

The second major aim of our study was to investigate and compare performance with *different types of displays*. Therefore, we created three types of displays, varying in abstractness and complexity. This was done for two reasons. First, we wanted to compare performance in the same task with different types of displays in order to test the robustness of our findings. Second, we wanted to compare performance with different displays in an attempt to develop useful displays for future experimental work on offside judgments. Each display showed the moment when a midfielder passed the ball to a forward of the same team, and we varied the spatial separation between the forward (F) and the defender (D). The first type of display involved colored triangles as players (Fig 1A; this simple and most artificial type of display was used in Experiments 1A/B). The second type of display involved clipart figures of soccer players (Fig 1B), and was used in Experiments 2A/B and 3. Finally, the third type of display consisted of custom-made photographs showing a game scene (Fig 1C); this was the most complex and natural type of display and was used in Experiment 4. In each case, participants were told from the outset that the displays are showing a soccer scene.

The use of static displays for studying offside judgments requires some justification because, in real soccer matches, assistant referees are confronted with a continuous stream of action and movement. Yet, we had two reasons for using static displays for our research. First, we wanted to isolate the most central (or basic) aspect in offside decisions. In particular, the application of the offside rule requires judging the relative spatial position of the forward (in relation to the second last defender) “*at the moment* the ball touches or is played by one of his team” (cf. [2]). And this is exactly the task we gave to our participants. Of course, as mentioned before, in real soccer matches, this basic task is complicated by additional variables such as the action of the defender, the speed of movement, and others. However, we found it useful to study offside judgments in the most basic variant of the task and, after having understood performance in this task, researchers could add other variables in order to increase the extrinsic validity of the results. Second, we also used static displays in order to increase the odds for detecting an *intrinsic* bias in our participants by minimizing the possible impact of movement-related effects on offside judgments as, for example, the flash-lag effect.

Judging offside in static displays is most likely to be an easier task than judging offside in dynamic scenes involving movement. Therefore, we did not know whether our task might be too easy, at least for some participants, even with short display durations. In particular, without masking, the retinal image of a display is available for some time after the display has disappeared. To prevent ceiling effects, we incorporated a second condition with backward masking of displays in Experiments 1A/B and 2A/B. A backward mask overwrites the retinal image of the display, allowing for more precise control of display duration and reducing the risk of ceiling effects in performance [28].

## Experiment 1A/B

Experiment 1A/B investigated offside judgments of laypersons when being confronted with simple visual displays. The displays used in Experiments 1A/B contained three colored triangles and a white circle on a green and gray background (cf. Fig 1A). One of the triangles and the white circle appeared in one half of the display and represented the midfielder passing the ball to his forward. The other half of the display contained a triangle in the same color as the midfielder, representing the forward, and a third triangle in a different color, representing the defender. The distance between the midfielder and the defender was fixed to 20 cm (21.8° of visual angle). This distance corresponds to a separation of 20 meters, when seen from assistant referee´s point of view with about 50 meters distance. We systematically varied the spatial separation between the forward and the defender in eleven steps from -5 mm to the left of the defender to +5 mm to the right of the defender. Each 1-mm step on the screen corresponds to a 10-cm step as seen from about 50 m distance. At each horizontal position, the forward could either appear in front of (and slightly below, 2mm) the defender, or behind (and slightly above, 2mm) the defender. Finally, each of these 22 situations could appear in the right half of the display, representing an attack of team A playing from left to right, or in the left half of the display, representing an attack of team B playing from right to left. In each of the 8 blocks of the experiment, the 44 displays were presented for 100 ms each in random order.

Participants took the role of an ad-hoc assistant referee who was responsible for the complete soccer field from the left to the right goal. The participants were informed that each display represented the moment at which a midfielder passed the ball to his forward, and their task was to judge whether the forward was offside, at that moment, or not. Consider a block of trials (representing one half of a soccer match) where the red team played from left to right and the blue team from right to left. In this block, the red forward was offside if he was closer to the right edge of the display than the blue defender. Similarly, the blue forward was offside if he was closer to the left edge of the display than the red defender. The offside and non-offside responses were mapped onto an upper and lower key, respectively.

There were two groups of participants. For one group, the displays were followed by an empty background in light gray (Experiment 1A). For the other group, the displays were followed by a pattern mask that was composed of small squares in different colors (Experiment 1B). The pattern mask was intended to overwrite the retinal image of the stimulus display, and would terminate the uptake of visual information [28].

The main goal of this experiment was to investigate the accuracy of offside judgments in laypersons as a function of the spatial separation between the forward and the defender in simple displays. Therefore, the dependent variable of major interest was the accuracy of offside judgments as a function of the spatial separation between forward and defender. Consistent with the findings of the investigation of the four-step spatial separation variation [10], we expected accuracy to be lowest at small distances between the forward and the defender. In addition, these authors found an asymmetry in that errors were higher when the forward was slightly behind the offside line than when he was slightly ahead of the offside line, which is consistent with the flash-lag hypothesis. If our use of static displays reduced the impact of the flash-lag effect, this asymmetry should not occur in our findings.

A second goal of our experiment was to determine whether a response bias exists in a sample of laypeople. We tried to minimize extrinsic sources for a response bias and, therefore, presented offside and non-offside situations with almost equal frequency. In fact, our experiments contained slightly more non-offside situations (6/11) than offside situations (5/11). The reason is that we wanted equal numbers of negative distance conditions (requiring a non-offside response) and positive distance conditions (requiring an offside response) in addition to the zero-distance condition (requiring a non-offside response, too). We did not provide error feedback in Experiment 1A/B. We computed the sensitivity index *d’* and the bias index *c* [20] separately for conditions without masking (Experiment 1A) and for conditions with masking (Experiment 1B). Although both measures are computed from the same statistics (i.e., hit rate and false-alarm rate), they are logically independent. The independence between *d’* and *c* results from the fact that any response bias increases or decreases both the hit and the false-alarm rate to the same degree, and hence does not alter the difference between the statistics, from which *d’* is computed [19, 20].

We also analyzed the *Reaction Times* (RTs) of judgments in order to assess the relationship between accuracy and RT. This is important because differences in accuracy (or error rates) might either reflect ‘true’ variations of task difficulty or deliberate changes of the speed-accuracy relationship. In the former case, accuracy and RT are positively correlated (i.e., low accuracy and long RT vs. high accuracy and short RT); in the latter case, accuracy and RT are negatively correlated (i.e., low accuracy and short RT vs. high accuracy and long RT) [29].

### Methods

#### Ethics statement

Experiments 1A/B and all subsequently reported experiments were carried out in accordance with the Helsinki Declaration of 1975 and the ethical guidelines of the German Society for Psychology. The Institutional Review Board at the Institute of Cognitive and Team/Racket Sport Research on the German Sport University Cologne has reviewed and approved the studies. The written informed consent was obtained from each participant before starting the experiment.

#### Participants

Forty-eight students (41 female, 7 male; mean age = 22.8 years. *SD* = 2.5) participated in Experiments 1A/B for course credit. The participants were randomly assigned to the no-mask (Experiment 1A; 20 females, 4 males; mean age: 22.4 years, *SD* = 2.4) and to the with-mask condition (Experiment 1B; 21 female, 3 male; mean age = 23.1 years, *SD* = 2.7). Participants were naïve with respect to the purpose of the study and reported normal (or corrected-to-normal) visual acuity. Before Experiments 1A/B and all subsequently reported experiments, participants were tested with selected plates from Ishihara’s test for color vision. All participants in this experiment and the subsequent studies reported no practical experience with refereeing soccer matches. However, we neither assessed their attitude towards soccer, nor the frequency of viewing soccer matches.

#### Apparatus and stimuli

Participants sat in a normally lit room in front of a 22-inch TFT color monitor. A computer program created with E-Prime software [30] controlled stimulus presentation and response registration. Participants responded by pressing the “down” or “up” key on a standard computer keyboard with the index finger of their right hand. We did not use a head-or-chin rest, but viewing distance was somewhat constrained by the fact that the keyboard was fixed to the desk at a distance of 50 cm from the screen.

The fixation point was a black plus sign (10 mm × 10 mm) presented at screen center. The stimulus pictures consisted of three colored triangles, representing the players, and a white circle, representing the ball, presented in front of a colored background (cf. Fig 1A). A complete stimulus picture had a width of 29 cm and a height of 18 cm. The lower half of the background was green (representing the playing field); the upper half of the background was light gray. The triangles represented a midfielder and a forward from team A, thus having the same color (e.g., blue), and a defender from team B, being in a different color (e.g. red). The triangles were 10 mm wide and 20 mm high (1.15° × 2.30°). The midfielder was presented at a left or right position approximately 4.5 cm from the outer edge of the stimulus picture. The defender was presented at the opposite side, with the distance between midfielder and defender being 200 mm (21.8°). The white circle (i.e. the ball) had a diameter of 5 mm and appeared at the more central side of the midfielder. The position of the forward (having the same color as the midfielder) was varied with regard to the position of the defender both on the horizontal and on the vertical axis. The horizontal position of the forward varied in 11 one-millimeter steps from 5 mm to the left to 5 mm to the right from the position of the defender. Subsequently, these steps are labeled from -5 (most extreme forward position towards own goal) to +5 (most extreme forward position towards opponent goal). Thus, at distance 0, the forward and the defender were presented at the same horizontal position (i.e. had the same distance from the midfielder). We varied the vertical position of the forward together with presenting him either in front of the defender (and 2 mm below), or behind (and 2 mm above) the defender. Hence, when the forward was presented below (i.e. in front of) the defender, the former occluded the latter to a large extent; when the forward was presented above (i.e. in front of) the defender, the latter occluded the former to a large extent (cf. Fig 1). The factorial combination of 2 vertical locations and 11 horizontal locations yielded 22 stimulus pictures with the same spatial midfielder-defender configuration.

In total, there were 88 different stimulus pictures that resulted from combining 2 possible colors of the attacking team (blue or red), 2 possible directions of attack (from left to right or from right to left), 2 vertical positions of the forward, and 11 horizontal positions of the forward. The 88 pictures were divided in two sets representing different half times of a soccer match. Picture set 1 contained those pictures where the blue team attacks from left to right and the red team attacks from right to left; picture set 2 contained those pictures where the red team attacks from left to right and the blue team attacks from right to left.

The pattern mask consisted of blue, gray, green, red and white squares, with a side length of 10 mm. The squares were arranged in 18 lines and 30 columns. Each line consisted of several sequences of blue, gray, green, red and white squares, with the arrangement being shifted by one place to the right from one line to the next. Hence, adjacent squares always had different colors.

#### Procedure

The experiment started with the presentation of the instructions on the computer screen. The first slide described the participants’ task, the offside rule, and the sequence of events in a typical trial. The participants were told they would see pictures with geometrical figures representing scenes from a soccer match. Moreover, they were asked to act as a referee and to decide, for each picture, whether the forward was offside or not. Then, two examples of stimulus pictures were presented with a short description on it. One picture showed an offside situation; the other picture showed a non-offside situation. Then a third slide informed participants about the S-R mapping: They were required to press the “up” key to offside situations and the “down” key for non-offside situations. Participants could view each instruction slide as long as they wished, and continued by keypress.

Having read the instructions, participants started a practice block with 44 trials (i.e. each trial contained a different picture). The practice block was followed by 8 experimental blocks with 44 trials each. Each block contained 44 different stimulus pictures that resulted from a factorial combination of 2 possible directions of attack (from left to right or from right to left), 2 vertical positions of the forward, and 11 horizontal positions of the forward. For half of the participants, the blue team played from left to right, and the red team played from right to left (picture set 1). The reverse was true for the other half of participants (picture set 2).

Each trial started with an empty gray screen for one second. Then the fixation point was presented for 500 ms, followed by the stimulus picture for 100 ms. In the no-mask condition (Experiment 1A), the stimulus picture was followed by another empty gray screen for two seconds. In the with-mask condition (Experiment 1B), the stimulus picture was followed by the pattern mask which was also presented for two seconds. During that two-second period, the computer registered the identity and latency (i.e. RT) of the participant’s keypress responses. There was no feedback regarding the correctness of this response. In each block, the 44 different stimulus pictures were presented in random order. After each block, a message told participants to take a short pause and to start the next block by pressing the space bar.

#### Design and data analysis

A 11 × 2 mixed factorial design with *Spatial Separation* (between forward and defender) as a within-subject factor, and *Masking* (with or without pattern mask) as a between-subject factor was used in the experiment. The dependent variables were the percentage of correct responses (PC) and reaction time (RT).

For each experiment, we also computed the sensitivity index *d’* and the response-bias index *c* from *z*-transformed hit and false-alarm rates [20]. Hit rates were calculated for each participant, by dividing the sum of correct offside judgments by all offside situations. False-alarm rates were calculated for each participant, by dividing the sum of false offside judgments (i.e. flag errors) by all non-offside situations. The index *d’* is computed from the formula *d’* = *z*(hit rate)–*z*(false-alarm rate) and as mentioned in the introduction represents the ability to discriminate offside from non-offside situations (varying between 0 and ~ 4.65) The response bias index *c* is computed from the formula *c* = -0.5 × (*z*[hit rate] + *z*[false-alarm rate]) and represents the participants preference for one or the other response. If *c* is statistically equivalent to 0, participants prefer neither response. A negative *c* arises when the false-alarm rate is higher than the *miss* rate, indicating a preference for the “offside” response. In contrast, a positive *c* arises when the false-alarm rate is lower than the *miss* rate, indicating a preference for the “non-offside” response [20].

Throughout this report, we corrected the results of *F* tests according to Huynh and Feldt [31] if Mauchly’s test of sphericity was significant. Moreover, we corrected the results of *t* tests if Levene’s [32] test of homogeneity was significant. To compare means within interactions, we used a variant of Tukey’s HSD method [33]. Concerning effect sizes, we report $\eta_{partial}^{2}$ for the results of *F* tests, and Hedges’s *g* for the results of *t* tests (e.g. [34]).

### Results

For each experiment, we removed the data from a participant if his or her performance was below 60% for at least one of the two easy conditions (i.e. -5 or +5). This applied to one participant from the no-mask condition (Experiment 1A; *PC* = 6% vs. sample *M* = 86%, *SD* = 21), and one participant from the with-mask condition (Experiment 1B; *PC* = 25% vs. sample *M* = 83%, *SD* = 16). Hence, the sample was reduced to 46 participants.

#### Percentage of Correct Responses

**Fig 2:**
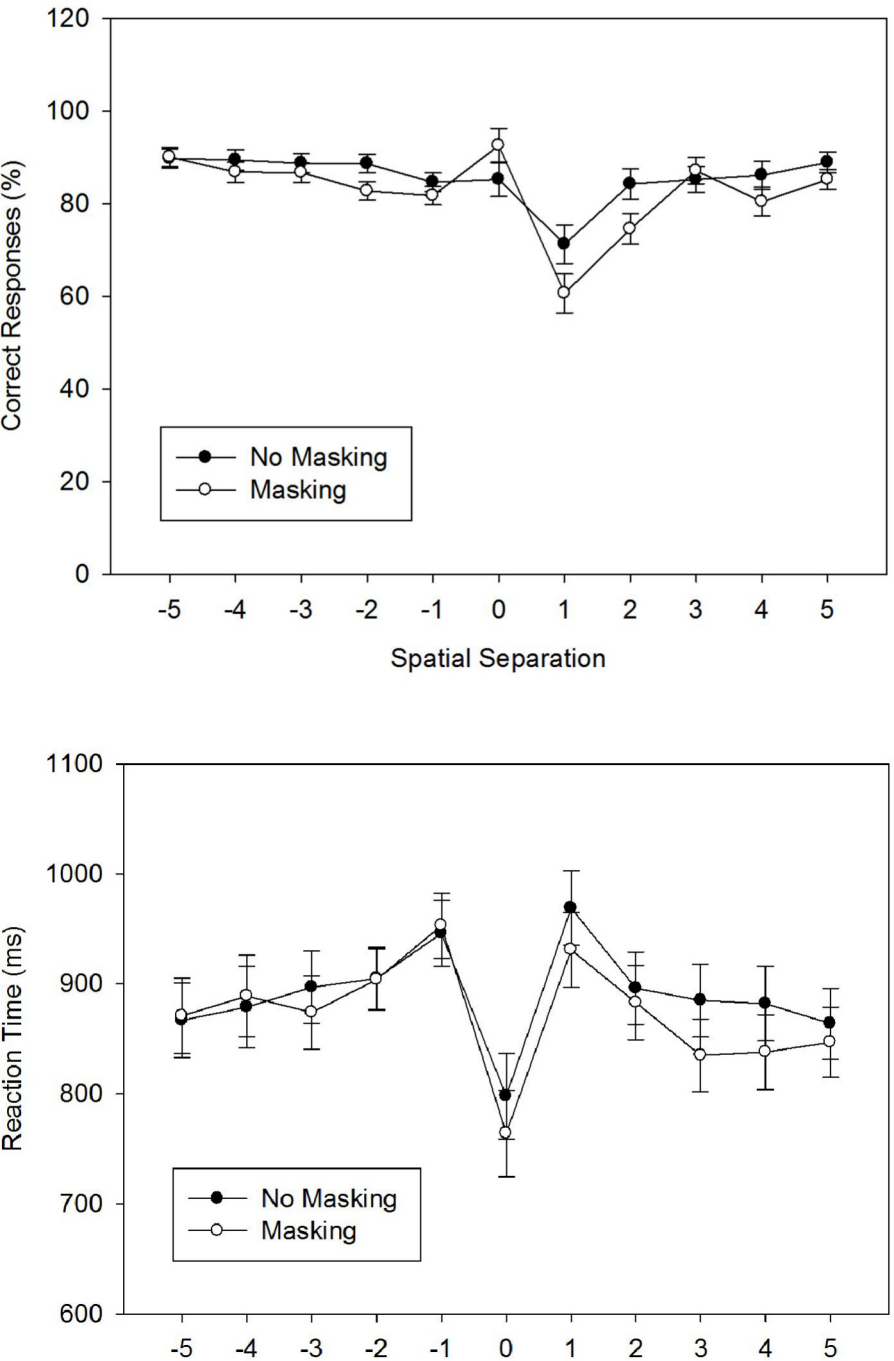
The figure shows the percentage of correct responses (Fig 2A) and the reaction times (Fig 2B) as a function of the spatial separation between forward and defender for two masking conditions (no masking vs. masking) in Experiment 1A/B. Error bars represent standard errors of the mean.

We first calculated the *PC* for each participant and each of the 22 experimental conditions, and entered these individual means into an 11 × 2 ANOVA with *Spatial Separation* as a within-participant factor and *Masking* as a between-participant factor. Fig 2A shows the arithmetic means across participants. The ANOVA revealed a significant main effect for *Spatial Separation*, *F*(4.65, 204.68) = 19.87, *MSE* = 223.56, *p* < .001, $\eta_{partial}^{2}$ = .31. The main effect of *Masking* was not significant, *F*(1, 44) = 2.09, MSE = 86.36, *p* = .155, $\eta_{partial}^{2}$ = .05, but the interaction of *Spatial Separation* and *Masking* was significant, *F*(4.65, 204.68) = 2.65, MSE = 223.56, *p* < .05, $\eta_{partial}^{2}$ = .06.

Concerning the main effect of *Spatial Separation*, repeated contrastsobtained a V-shaped pattern with a minimum at level +1. Repeated contrasts compare the mean for each factor level to the subsequent mean [35]. This procedure is appropriate if factor levels are ordered on a common dimension (e.g., horizontal distance). In particular, there were marginally significant reductions from level -3 to level -2, *F*(1, 44) = 3.75, *p* = .059, $\eta_{partial}^{2}$ = .08, and from level -2 to level -1, *F*(1, 44) = 3.17, *p* = .082, $\eta_{partial}^{2}$ = .07, and a marginally significant increase from level -1 to level 0, *F*(1, 44) = 3.90, *p* = .055, $\eta_{partial}^{2}$ = .08. Moreover, there was a significant decrease of PC from level 0 to level +1, *F*(1, 44) = 34.26, *p* < .001, $\eta_{partial}^{2}$ = .44, and a significant increase from level +1 to level +2, *F*(1, 44) = 64.11, *p* < .001, $\eta_{partial}^{2}$ = .59. Finally, there was again a significant increase from level +4 to level +5, *F*(1, 44) = 8.81, *p* < .01, $\eta_{partial}^{2}$ = .17. Concerning the interaction, results showed that masking significantly decreased PC for level +1, *g* = 0.53, and for level +2, *g* = 0.62 (Tukey’s HSD = 9.78).

#### Reaction Time

We first calculated the RTs of correct responses for each participant and each experimental condition, and entered these individual means into an 11 × 2 ANOVA with *Spatial Separation* as a within-participant factor and *Masking* as a between-participant factor. Fig 2B shows the arithmetic means across participants. The ANOVA revealed a significant main effect for *Spatial Separation*, *F*(5.00, 220.12) = 12.05, *MSE* = 16683.47, *p* < .001, $\eta_{partial}^{2}$ = .22. Neither the main effect of *Masking* nor the interaction of *Spatial Separation* × *Masking* were significant, both *F* < 1.0.

Concerning the main effect of *Spatial Separation*, repeated contrasts revealed an M-shaped RT function with a minimum at level 0. In particular, RT significantly increased from level -2 to level -1, *F*(1, 44) = 11.23, *p* < .01, $\eta_{partial}^{2}$ = .20, decreased from level -1 to level 0, *F*(1, 44) = 42.88, *p* < .001, $\eta_{partial}^{2}$ = .49, increased again from level 0 to level 1, *F*(1, 44) = 37.87, *p* < .001, $\eta_{partial}^{2}$ = .46, and then decreased again from level 1 to level 2, *F*(1, 44) = 17.75, *p* < .001, $\eta_{partial}^{2}$ = .29, and from level 2 to level 3, *F*(1, 44) = 6.24, *p* < .05, $\eta_{partial}^{2}$ = .12.

**Table 2:** Sensitivity indices *d’* and response-bias indices *c* as observed in different conditions of Experiments 1–4. All reported values are significantly larger than zero. A positive c reflects a preference for the “non-offside” response, which is associated with a preponderance of non-flag errors (in offside situations).

	*d’*	c
**Experiment 1a –No Masking**	2.28	0.19
**Experiment 1b –Backward Masking**	1.92	0.24
**Experiment 2a –No Masking**	2.25	0.25
**Experiment 2b –Backward Masking**	1.48	0.11
**Experiment 3 (Feedback)**	1.93	0.19
**Experiment 4 (Photos)**	1.22	0.30
***Mean***	1.85	0.21

#### Signal-Detection Analysis

The sensitivity index *d’* was 2.28 (SD = 1.31) for the no-mask condition, and 1.92 (SD = 0.77) for the with-mask condition (see Table 2). Each index was significantly different from 0, both *t*s(23) > 8.5, both *p*s < .001, both *g*s > 1.70. Consistent with the results in PC, masking did not significantly decrease *d’*, *t*(46) = 1.18, *p* = .25, *g* = 0.35. The bias measure *c* was 0.19 (SD = 0.20) for the no-mask condition, and 0.24 (SD = 0.20) for the with-mask condition. Each index was significantly different from 0, both *t*s(23) > 4.5, both *p*s < .001, both *g*s > 0.90, indicating a significant preference for the “non-offside” response. The bias measure did not differ between the no-mask and the with-mask condition, *t*(46) = -0.88, *p* = .39, *g* = 0.25.

### Discussion

Experiment 1A/B investigated offside judgments with simple static displays. Concerning overall performance, perceptual sensitivity was good for both masking conditions across spatial distances. This means that participants were able to discriminate offside from non-offside situations well above chance. The bias measure *c* was significantly positive for both masking conditions, indicating a preference for the non-offside response, which is associated with a preponderance of non-flag errors.

There was a strong impact of spatial separation on performance. In particular, when compared to large negative and large positive separations, performance suffered between level -3 and level +3, with a minimum at level +1 (the first offside situation). Note that the asymmetric shape of the *PC* function, with its minimum at level +1 and not at level 0, is a direct consequence of the bias in favor of the non-offside response. The pattern was quite similar in both masking conditions, with a notable exception at level 0, where accuracy was maximal with a mask.

Finally, there was a positive relationship between accuracy and RTs: RTs generally increased when accuracy decreased. Hence, both measures consistently signaled the difficulty of the condition, and there is no indication of a speed-accuracy tradeoff. This is also true for level 0, where there is a maximum in accuracy and a minimum in RT, suggesting that this condition is unusually simple, when compared to the most similar conditions. One question for the subsequent experiments is whether this finding represents a peculiarity of the displays used in Experiment 1 or not.

## Experiments 2A/B

Experiment 2A/B further investigated the spatial resolution in offside judgments of laypersons, but with a new set of displays. The displays in Experiment 2A/B contained three clipart figures of soccer players on a green and gray background (cf. Fig 1B). The clipart players were arranged in exactly the same way as the geometric shapes in the displays from Experiment 1A/B. Except for the displays, all other aspects of the methods used in Experiment 2A/B were similar to the methods used in Experiment 1A/B.

Our main question was whether performance with the new set of displays in Experiment 2A/B would or would not be similar to performance with the displays used in Experiment 1A/B. In particular, we were interested in whether we would replicate the response bias in favor of the non-offside response, and the impact of spatial separation on both PC and RTs.

### Methods

#### Participants

Forty-seven students (15 female, 33 male; mean age = 23.3 years, *SD =* 3.3) participated in Experiment 2A/B for course credit. Twenty-four were randomly assigned to the no-mask condition (Experiment 2A; 8 females, 16 males; mean age: 22.9 years, SD = 2.8) and twenty-three to the with-mask condition (Experiment 2B; 7 female, 16 male; mean age = 23.7 years, SD = 3.8). Participants were naïve with respect to the purpose of the study and reported normal (or corrected-to-normal) visual acuity.

#### Apparatus and stimuli

The apparatus, the fixation point and the pattern mask used in Experiment 1A/B were also used in Experiment 2A/B. Instead of colored triangles, however, we used different clipart characters of soccer players as the midfielder, the forward, and the defender (cf. Fig 1B). All clipart characters were 30 mm high and 21–24 mm wide.

Similar to Experiment 1, we produced 88 different stimulus pictures from combining 2 possible colors (blue or red), 2 possible directions of attack, 2 vertical positions of the forward (forward below/in front defender, forward above/behind defender), and 11 horizontal positions of the forward. Again, the pictures were divided in two sets corresponding to different half-times of a soccer match.

#### Procedure

The procedure and trial structure of Experiment 2A/B were the same as in Experiments 1A/B. Moreover, as in these experiments, we counterbalanced picture set (set 1: blue team playing from left to right and red team playing from right to left; set 2: red team playing from left to right and blue team playing from right to left) and masking condition (Experiment 2A: no-mask; Experiment 2B: with-mask) across participants.

#### Design and data analysis

A 11 × 2 mixed factorial design with *Spatial Separation* (between forward and defender) as a within-subject factor, and *Masking* (with or without pattern mask) as a between-subject factor was used in the experiment. The dependent variables were PC and RT. Moreover, for each masking condition, we also computed the sensitivity index *d’* and the bias index *c*.

### Results

Again, we removed participants if their performance for at least one of the two easy conditions (i.e. -5 or +5) was below 60%. This applied to two participants in the no-mask condition (Experiment 2B; PCs of 44% and 34%; sample *M* = 81%, *SD* = 17). Hence, the sample was reduced to 45 participants (24 participants in Experiment 2A; 21 participants in Experiment 2B).

#### Percentage of Correct Responses

**Fig 3:**
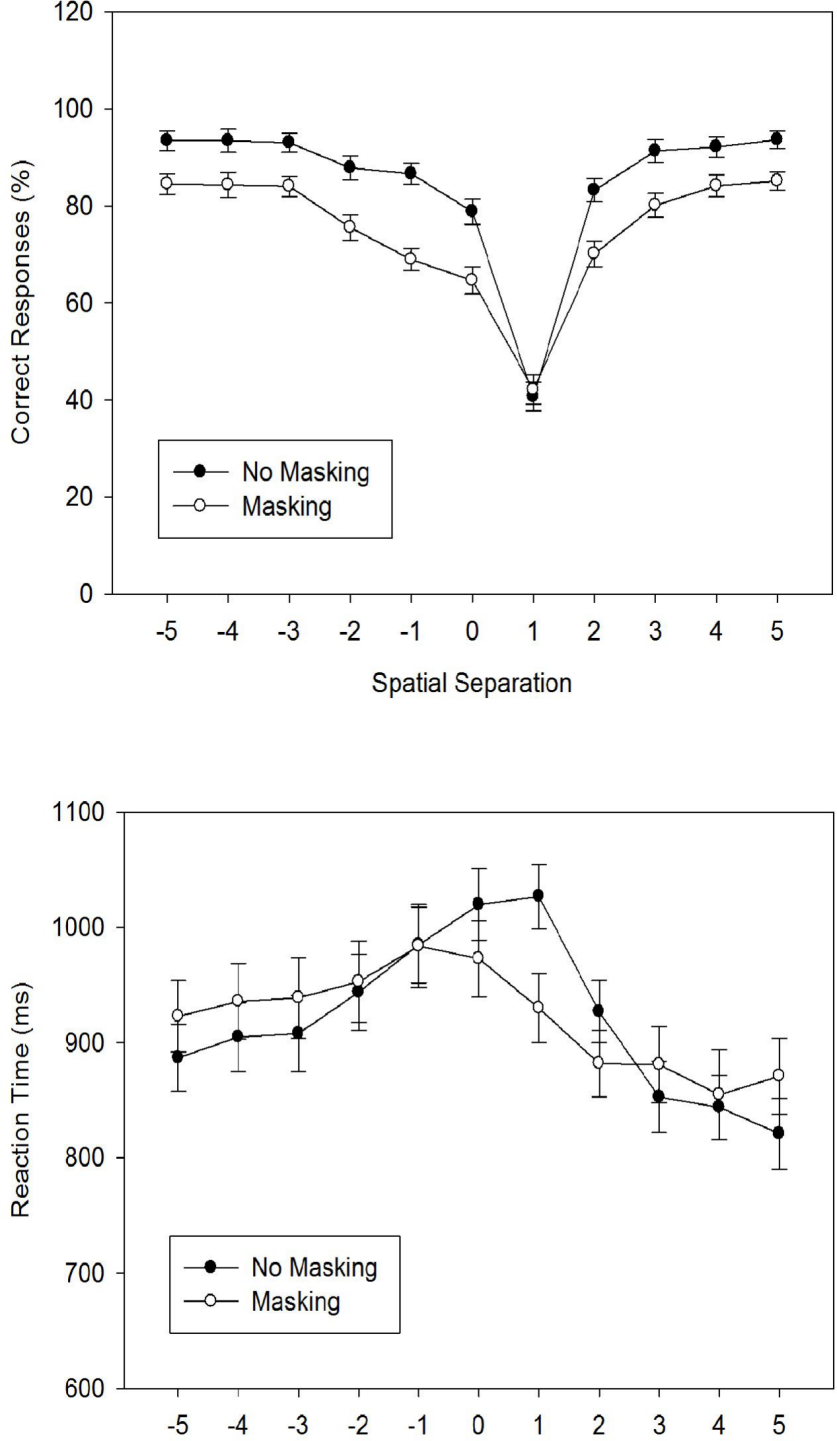
The figure shows the percentage of correct responses (Fig 3A) and the reaction times (Fig 3B) as a function of the spatial separation between forward and defender for two masking conditions (no masking vs. masking) in Experiment 2A/B. Error bars represent standard errors of the mean.

We calculated *PC* for each participant and each of the 22 experimental conditions, and entered these individual means into an 11 × 2 ANOVA with *Spatial Separation* as a within-participant factor and *Masking* as a between-participant factor. Fig 3A shows the arithmetic means across participants. The ANOVA revealed a significant main effect for *Spatial Separation*, *F*(4.46, 191.82) = 109.69, *MSE* = 182.55, *p* < .001, $\eta_{partial}^{2}$ = .72. The main effect of *Masking* was also significant, *F*(1, 43) = 21.17, MSE = 53.41, *p* < .001, $\eta_{partial}^{2}$ = .33, indicating higher *PC* values without masking (*M* = 84.87%; *SD* = 5.34) than with masking (*M* = 74.82%, *SD* = 9.06). Finally, the interaction of *Spatial Separation* and *Masking* was significant, *F*(4.46, 191.82) = 3.23, MSE = 182.55, *p* < .05, $\eta_{partial}^{2}$ = .07.

Concerning the main effect of *Spatial Separation*, repeated contrasts revealed a V-shaped pattern of *PC* with a minimum at level +1. In particular, there were significant reductions of *PC* for all four steps from level -3 to level +1, all *F*s(1, 43) > 9.00, all *p*s < .01, all $\eta_{partial}^{2}$ > .17. Conversely, there were significant increases of *PC* for all three steps from level +1 to level +4, all *F*s(1, 43) > 4.50, all *p*s < .05, all $\eta_{partial}^{2}$ > .09. Concerning the interaction, results showed that masking significantly decreased PC for all levels (all *g* > 0.65) except for levels +1 and +4 (Tukey’s HSD = 8.47).

#### Reaction Time

We first calculated the RTs of correct responses for each participant and each experimental condition, and entered these individual means into an 11 × 2 ANOVA with *Spatial Separation* as a within-participant factor and *Masking* as a between-participant factor. Fig 3B shows the arithmetic means across participants. The ANOVA revealed a significant main effect for *Spatial Separation*, *F*(5.49, 235.96) = 21.97, *MSE* = 10,448.96, *p* < .001, $\eta_{partial}^{2}$ = .34. The main effect of *Masking* was not significant, *F*(1, 43) < 1.0, but the interaction of *Spatial Separation* × *Masking* was significant, *F*(5.49, 235.96) = 3.88, *MSE* = 10,448.96, *p* < .01, $\eta_{partial}^{2}$ = .08.

Concerning the main effect of *Spatial Separation*, repeated contrasts revealed a bell-shaped RT function with its maximum at level 0. In fact, there was a marginally significant RT increase from level -5 to level -4, *F*(1, 43) = 3.41, *p* = .072, $\eta_{partial}^{2}$ = .07, and significant RT increases from level -3 to level -2, *F*(1, 43) = 6.47, *p* < .05, $\eta_{partial}^{2}$ = .13, and from level -2 to level -1, *F*(1, 43) = 7.31, *p* < .05, $\eta_{partial}^{2}$ = .15. Conversely, RT significantly decreased from level +1 to level +2, *F*(1, 43) = 25.68, *p* < .001, $\eta_{partial}^{2}$ = .37, and from level +2 to level +3, *F*(1, 43) = 13.83, *p* < .01, $\eta_{partial}^{2}$ = .24, and marginally so from level +3 to level +4, *F*(1, 43) = 3.24, *p* = .079, $\eta_{partial}^{2}$ = .07. Finally, concerning the interaction, results showed that masking significantly decreased RT for level +1, *g* = 0.70, only (Tukey’s HSD = 71 ms).

#### Signal-Detection Analysis

The sensitivity index *d’* was 2.25 (*SD* = 0.50) for the no-mask condition (Experiment 2A), and 1.48 (*SD* = 0.58) for the with-mask condition (Experiment 2B; cf. Table 2). Both indices were significantly different from 0, *t*(23) = 22.23, *p* < .001, *g* = 4.51, and *t*(20) = 11.78, *p* < .001, *g* = 2.55. Consistent with the results in PC, masking significantly decreased *d’*, *t*(43) = 4.86, *p* < .001, *g* = 1.43. The bias measure *c* was 0.25 (*SD* = 0.18) for the no-mask condition, and 0.11 (*SD* = 0.22) for the with-mask condition. Each index was significantly different from 0, *t*(23) = 6.96, *p* < .001, *g* = 1.39, and *t*(20) = 2.35, *p* < .001, *g* = 0.50, indicating significant preferences for the “non-offside” response. The bias was larger for the no-mask condition, *t*(43) = 2.44, *p* < .05, *g* = 0.70.

### Discussion

Experiment 2A/B used more realistic displays (depicting clipart soccer players) than Experiment 1A/B to investigate offside judgments in laypersons. Concerning overall performance, perceptual sensitivity was good for both masking conditions (*d’* = 2.25 and 1.48). However, in contrast to Experiment 1A/B, masking decreased *d’* in Experiment 2A/B. Replicating Experiment 1A/B, a positive bias measure for both masking conditions indicated a preference for the non-offside response, which is associated with a preponderance of non-flag errors.

The spatial separation between forward and defender had a strong effect on accuracy. In general, accuracy decreased from level -3 to a minimum at level +1 (the first offside situation), and then increased again until level +4. Masking reduced accuracy for all levels, except for level +1. When compared to Experiment 1A/B, we replicated the performance minimum at level +1. However, spatial separation seems to have had a larger impact in Experiment 2A/B than in Experiment 1A/B, and the recovery of accuracy at level 0 in Experiment 1A/B was absent in Experiment 2A/B. In contrast, the accuracy function in Experiment 2A/B had an almost symmetrical V shape with its minimum at level +1.

Finally, as in Experiment 1A/B, RTs generally increased when accuracy decreased. Hence, there was again no indication for a speed-accuracy tradeoff in Experiment 2A/B. As for accuracy, the RT functions from both experiments markedly differed at level 0: Whereas accuracy was high and RTs short for level 0 in Experiment 1A/B, accuracy was low and RT long for level 0 in Experiment 2A/B. This difference turned the M-shaped RT function from Experiment 1A/B into a bell-shaped RT function in Experiment 2A/B.

Thus, both experiments revealed a significant effect of spatial separation on offside judgments, with a minimum of performance at level +1, and this pattern was associated with a preponderance of non-flag errors in both experiments. Yet, there were also differences. The first difference was that overall performance was worse in Experiment 2A/B, suggesting that the clipart figures are more difficult to judge than triangles. This difference might have emerged from the fact that triangles have regular (linear) shapes, whereas clipart figures have irregular shapes. Hence, with small distances between forward and defender, only parts of the clipart forward are shifted towards one side of the defender, whereas the whole side of a triangle forward is shifted towards one side of the defender. The second difference was that the spatial separation between forward and defender had a larger impact in Experiment 2A/B. The third difference concerned performance at level 0, which was maximal in Experiment 1A/B, but close to minimum in Experiment 2A/B. Hence, in contrast to the clipart figures, presenting two triangles at the same horizontal location (level 0) creates a particularly salient configuration that is easily identified by the participants.

## Experiment 3

The purpose of Experiment 3 was to investigate the impact of feedback on offside judgments in laypersons. As mentioned in the introduction, three studies suggest that feedback can influence the quality of referee decisions [25–27], but it is still unclear whether a single session with feedback is sufficient to reduce biases in offside judgments. Therefore, in our study we repeated the no-mask and no-feedback condition from Experiment 2A with a new group of participants, but now the computer gave feedback after each response. In particular, in Experiment 3, each correct response was followed by the message “Correct!” on the screen, whereas each wrong response was followed by the message “Wrong!” on the screen. The question was how consistent feedback through the course of an experiment (consisting of 44 practice trials and 360 experimental trials) would affect offside judgments in laypersons. We were particularly interested in, first, whether feedback would reduce or eliminate the response bias observed in Experiments 1A/B and 2A/B and, second, whether feedback might increase perceptual sensitivity. In order to evaluate the effects of feedback, we planned to compare the results of Experiment 3 to the results of Experiment 2A because these experiments were similar except for the feedback provided in Experiment 3. There was no masking condition in Experiment 3 because masking did not have large effects in Experiments 1A/B and 2A/B.

### Methods

#### Participants

Thirty students (20 female, 10 male; mean age = 23.4 years, SD = 3.1.) participated in Experiment 3 for course credit. None of them had participated in Experiment 1 or 2. Participants were naïve with respect to the purpose of the study and reported normal (or corrected-to-normal) visual acuity.

#### Apparatus and stimuli

The apparatus and stimulus material from Experiment 2A was also used in Experiment 3, with the following exceptions. The words “Correct!” and “Wrong!” were used as feedback stimuli in Experiment 3. Both words were presented in courier font size 18 at the screen center. The word “Correct” was presented in blue color, the word “Wrong” was presented in red color.

#### Procedure

The procedure and trial structure of Experiment 3 were the same as in Experiment 2A, except for the fact that a feedback message was presented in each trial for one second after the blank response interval of 2 seconds. Note that both correct responses and wrong responses received distinct and reliable feedback.

#### Design and data analysis

A 11 × 2 mixed factorial design with *Spatial Separation* (between forward and defender) as a within-subject factor, and *Feedback* (with or without) as a between-subject factor was used in Experiment 3. The dependent variables were PC and RT. Moreover, for both feedback conditions, we also computed the sensitivity index *d’* and the response-bias index *c*.

### Results

Again, we removed participants if their performance for at least one of the two easy conditions (i.e. -5 or +5) was below 60%. This was the case for two participants from Experiment 3 (*PC*s of 31% and 38% for level +5; sample *M* = 86%, *SD* = 16). Hence, 28 participants contributed data to Experiment 3.

#### Percentage of Correct Responses

**Fig 4:**
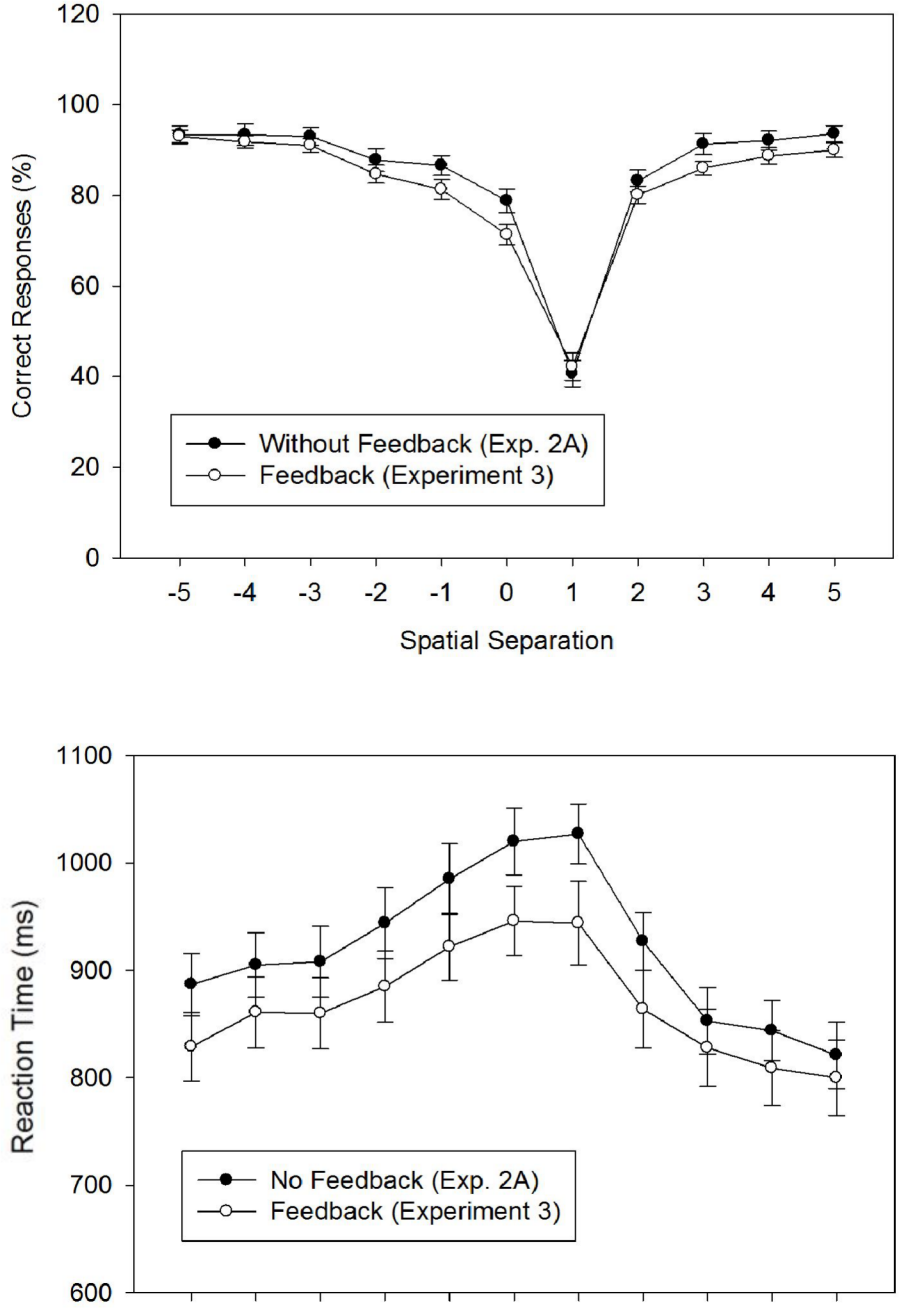
The figure shows the percentage of correct responses (Fig 4A) and the reaction times (Fig 4B) as a function of the spatial separation between forward and defender for two feedback conditions. In fact, no-feedback condition is identical to the no-masking condition of Experiment 2A; data for the feedback condition were collected in Experiment 3. Error bars represent standard errors of the mean.

We calculated *PC* for each participant and each of the 22 experimental conditions, and entered these means into an 11 × 2 ANOVA with *Spatial Separation* as a within-participant factor and *Feedback* (no feedback: Experiment 2A; feedback: Experiment 3) as a between-participant factor. Fig 4A shows the arithmetic means across participants. The ANOVA revealed a significant main effect for *Spatial Separation*, *F*(4.10, 205.13) = 182.89, *MSE* = 153.86, *p* < .001, $\eta_{partial}^{2}$ = .79. The main effect of *Feedback* was marginally significant, *F*(1, 50) = 3.68, MSE = 35.21, *p* = .061, $\eta_{partial}^{2}$ = .07, representing the unexpected finding that PC values were lower with feedback (*M* = 81.71%; *SD* = 6.40) than without feedback (*M* = 84.87%, *SD* = 5.34). The interaction of *Spatial Separation* × *Feedback* was not significant, *F*(4.10, 205.13) = 1.23, MSE = 153.86, *p* = .30, $\eta_{partial}^{2}$ = .02.

Concerning the main effect of *Spatial Separation*, repeated contrasts revealed a V-shaped pattern with a minimum at level +1. In particular, there were significant reductions of *PC* for all four steps from level -3 to level +1, all *F*s(1, 50) > 4.10, all *p*s < .05, all $\eta_{partial}^{2}$ > .07. Conversely, there were significant increases of *PC* for the two steps from level +1 to level +2, *F*(1, 50) = 409.75, *p* < .001, $\eta_{partial}^{2}$ = .89, and level +2 to level +3, *F*(1, 50) = 32.90, *p* < .001, $\eta_{partial}^{2}$ = .40, and a marginally significant increase of *PC* for the step from level +3 to level +4, *F*(1, 50) = 3.85, *p* = .055, $\eta_{partial}^{2}$ = .07.

#### Reaction Time

We first calculated the RTs of correct responses for each participant and each experimental conditions, and entered these individual means into an 11 × 2 ANOVA with *Spatial Separation* as a within-participant factor and *Feedback* as a between-participant factor. Fig 4B shows the arithmetic means across participants. The ANOVA revealed a significant main effect for *Spatial Separation*, *F*(4.99, 249.63) = 36.30, *MSE* = 51,514.82, *p* < .001, $\eta_{partial}^{2}$ = .42. The main effect of *Feedback*, *F*(1, 50) = 1.69, *MSE* = 20,504.45, *p* = .199, $\eta_{partial}^{2}$ = .03, and the interaction of *Spatial Separation* × *Feedback*, *F* < 1.0, were not significant.

Concerning the main effect of *Spatial Separation*, repeated contrasts revealed a bell-shaped RT function with a maximum across levels 0 and +1. In fact, RT significantly increased from level -5 to level -4, from level -3 to level -2, from level -2 to level -1, and from level -1 to level 0, all *F*s(1, 50) > 4.50, all *p*s < .05, all $\eta_{partial}^{2}$ > .08. Conversely, RT significantly decreased from level +1 to level +2, *F*(1, 50) = 43.96, *p* < .001, $\eta_{partial}^{2}$ = .47, and from level +2 to level +3, *F*(1, 50) = 53.72, *p* < .001, $\eta_{partial}^{2}$ = .52, and marginally so from level +4 to level +5, *F*(1, 50) = 3.88, *p* = .054, $\eta_{partial}^{2}$ = .07.

#### Signal-Detection Analysis

The sensitivity index *d’* was 1.93 (*SD* = 0.52) for the feedback condition, which was significantly above 0, *t*(27) = 19.66, *p* < .001, *g* = 3.71 (cf. Table 2). The bias measure *c* was 0.19 (*SD* = 0.18) for the feedback condition, which was also significantly larger than 0, *t*(27) = 5.69, *p* < .001, *g* = 1.06, indicating a preference for the “non-offside” response. When compared to the corresponding values of the no-feedback (no-mask) condition from Experiment 2A (*d’* = 2.25, *c* = 0.25), *d’* was smaller with feedback, *t*(50) = 2.27, *p* < .05, *g* = 0.63, while *c* values did not differ, *t*(50) = 1.35, *p* = .18, *g* = 0.22.

### Discussion

The purpose of Experiment 3 was to investigate the effects of simple feedback on offside judgments in laypersons. Therefore, we compared performance observed in Experiment 3 to the results of a no-feedback (and without feedback) condition of Experiment 2A. At first sight, the results of Experiment 3 appear strikingly similar to the results of Experiment 2A in qualitative terms (cf. Fig 4). Moreover, providing simple feedback did not reduce the response bias in favor of the non-offside response, underscoring the robustness of the bias.

However, there were some modest effects of feedback on performance, albeit in unexpected directions. In particular, *PC* was marginally lower and *d’* was significantly lower with feedback than without. When considered in isolation, these results might be attributed to a sampling error, or a detrimental effect of feedback in our task. However, a somewhat different explanation emerges when the accuracy effects are related to the numerical trend for shorter *RT*s (*F* = 1.69, *p* = .199) with feedback. In particular, decreased accuracy and shorter RTs with feedback, compared to the no-feedback condition, suggest that feedback did not disrupt performance in general, but led participants to adopt a laxer speed-accuracy relationship. A possible reason for adopting a laxer criterion with feedback might be that positive feedback in most of the trials (i.e., on average, in 82% of the trials) induced a positive mood in our participants that was more positive than that of the participants in Experiment 2 without feedback. In fact, previous research has shown that positive mood can lead to faster (and less accurate) decisions than neutral or negative mood (e.g. [36, 37]). However, this is a post-hoc hypothesis, which could be tested in subsequent experiments by explicitly probing participants’ mood (after each block of trials).

In sum, the results of Experiment 3 show that simply providing response feedback neither reduces the response bias nor improves the accuracy of offside judgments in laypersons. This pattern parallels that of another effect in which participants are biased to report a target as further along its anticipated trajectory (e.g., representational momentum; [39]), and in which feedback doesn’t seem to affect the response bias. Possibly, positive effects of feedback could be observed with more practice [26, 27].

## Experiment 4

Experiment 4 complemented Experiments 1A/B and 2A/B by investigating the spatial resolution in offside judgments of laypersons with a more realistic set of displays. Therefore, in Experiment 4, we used photographs showing a potential offside situation from a soccer match involving three players: a midfielder with the ball, a forward and a defender. We arranged the depicted scenes in a way that they matched the displays used in Experiments 1–3 as closely as possible. Except for the displays, all other aspects of the methods used in Experiment 4 were similar to the methods used in Experiments 1A and 2A (no-mask conditions). Hence, there was neither masking nor feedback in Experiment 4.

### Methods

#### Participants

Twenty-nine students (24 female, 4 male; mean age = 24.9 years, *SD* = 4.4) participated in Experiment 4 for course credit. Participants were naïve with respect to the purpose of the study and reported normal (or corrected-to-normal) visual acuity.

#### Apparatus and stimuli

The apparatus used for Experiment 4 was similar to that used for Experiments 1–3. However, the stimulus pictures were new: Instead of presenting artificial displays, we now presented real photographs of soccer situations involving three players: a midfielder and a forward from team A, and a defender from team B. In particular, we produced a photographic version for each of the 88 stimulus displays used in the previous experiments (cf. Fig 1C). Photographs were taken in four sessions on a sports ground at German Sport University Cologne. The models were three male Sports students at the German Sport University, who provided informed consent to the use of their photographs as stimulus materials in a subsequent laboratory experiment and for publication. The characters depicted on the photographs were 20 mm high, but differed in posture (and width) across photographs because the pictures were taken from dynamic game situations (10 photographs per second). In particular, we took sequences of pictures from scenes in which the midfielder passes the ball to the forward while the forward moved from a position in front of the defender to a position behind him. We produced sequences of pictures for each combination of 2 possible colors of the attacking team (blue vs. red), 2 possible directions of attack (from left to right vs. from right to left) and 2 vertical positions of the forward (below / in front of defender vs. above / behind defender). Finally, from these sequences, we selected 11 pictures in such a way that the horizontal distance between the forward and the defender varied in 11 equally-spaced steps that corresponded to approximately 1mm on the photograph.

#### Procedure

The procedure and trial structure of Experiment 4 were the same as in the no-mask condition of Experiment 2A, except that the stimulus pictures were presented for 250ms instead of 100ms. The duration of stimulus presentation was increased because we expected judging offside from the photographs in Experiment 4 a more difficult task than judging offside from the clipart displays in Experiments 2A/B and 3. In particular, figure-ground segmentation should be more difficult in Experiment 4 (than in Experiments 2A/B and 3) because of the heterogeneous background. Moreover, judgments of relative positions of players should be more difficult in Experiment 4 (than in Experiments 2A/B and 3) because of more variance in player posture and player configurations across displays. There was no backward masking because we did not expect ceiling effects in performance with the displays used in Experiment 4. Finally, participants received no feedback regarding the correctness of their responses.

#### Design and data analysis

A one-factorial design with *Spatial Separation* (between forward and defender) as a within-subject factor with 11 levels was used in Experiment 4. The dependent variables were PC and RT. In addition we computed the sensitivity index *d’* and the bias index *c* across spatial separations.

### Results

Again, we removed participants if their performance for at least one of the two easy conditions (i.e. -5 or +5) was below 60%. This applied to five participants from Experiment 4. In particular, for level -5, mean *PC* for the five participants was 35.6%, compared to a sample mean of 85.4% (*SD* = 9.11). Hence, 24 participants contributed data to Experiment 4.

#### Percentage of Correct Responses

**Fig 5:**
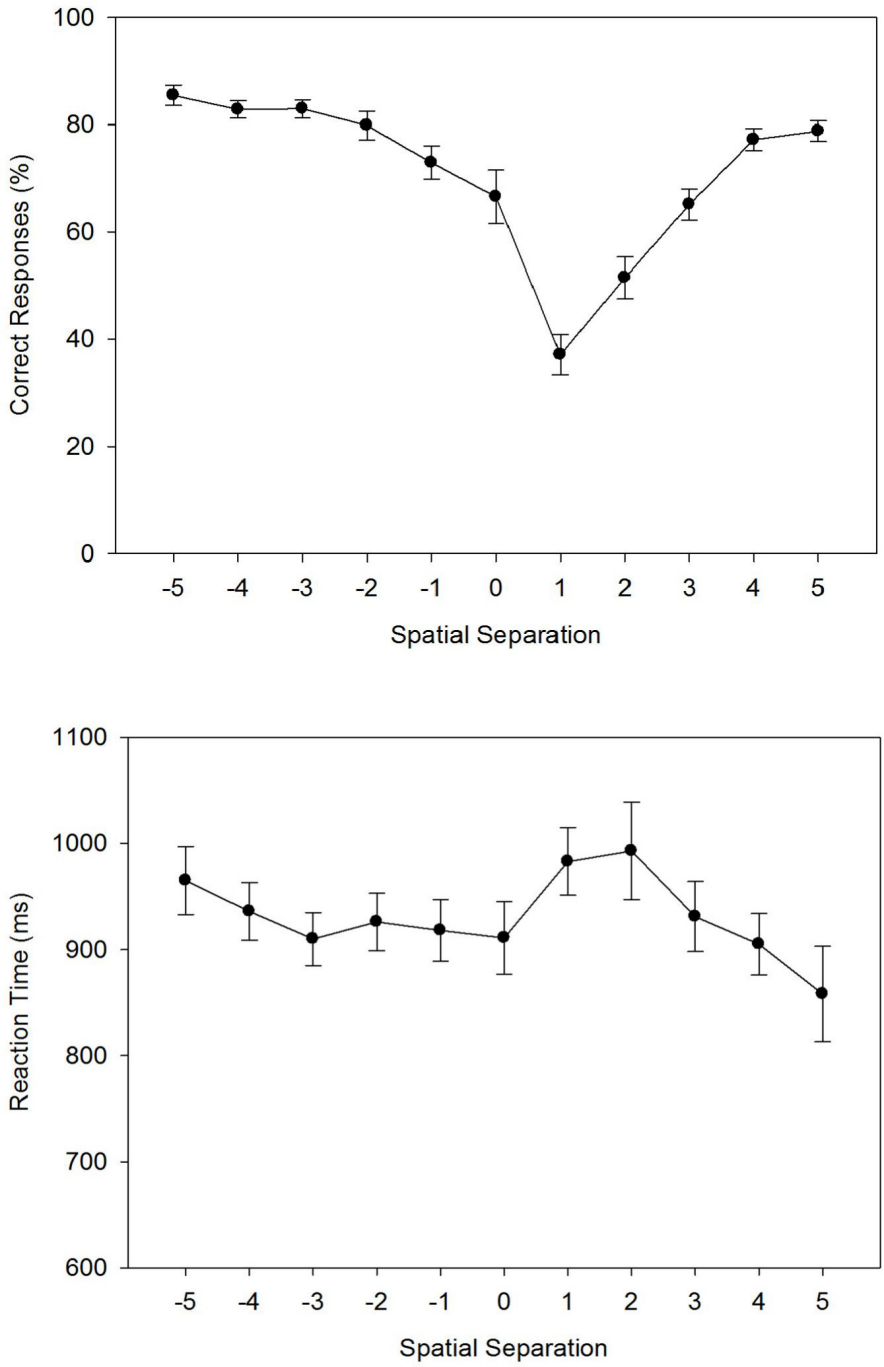
The figure shows the percentage of correct responses (Fig 5A) and the reaction times (Fig 5B) as a function of the spatial separation between forward and defender as observed in Experiment 4. Error bars represent standard errors of the mean.

We entered *PC* means into a one-factorial ANOVA with *Spatial Separation* as a within-subject factor. Fig 5A shows the arithmetic means across participants. The ANOVA revealed a significant main effect for *Spatial Separation*, *F*(2.21, 50.79) = 27.69, *MSE* = 885.56, *p* < .001, $\eta_{partial}^{2}$ = .55. Repeated contrasts revealed a V-shaped pattern of *PC* as a function of spatial separation with a minimum at level +1. In particular, there were significant decreases of *PC* for all three steps from level -2 to level +1, all *Fs*(1, 23) > 4.30, all *p*s < .05, all $\eta_{partial}^{2}$ > .16. Conversely, there were significant increases of *PC* for all three steps from level +1 to level +4, all *F*s(1, 23) > 17.00, all *p*s < .001, all $\eta_{partial}^{2}$ > .42.

#### Reaction Time

We first calculated the RTs of correct responses for each participant and experimental condition, and entered these individual means into a one-factorial ANOVA with *Spatial Separation* as a within-subject factor. Fig 5B shows the arithmetic means across participants. The ANOVA revealed a significant main effect for *Spatial Separation*, *F*(5.06, 116.33) = 2.45, *MSE* = 28,899.05, *p* < .05, $\eta_{partial}^{2}$ = .10. The RT function, however, appears rather flat and, consequently, repeated contrasts detected only one significant RT decrease from level -5 to level -4, *F*(1, 23) = 5.08, *p* < .05, $\eta_{partial}^{2}$ = .18; all other *F*s(1, 23) < 3.1, all other *p*s > .09, all $\eta_{partial}^{2}$ < .12.

#### Signal-Detection Analysis

The sensitivity index *d’* was 1.22 (*SD* = 0.33), which was significantly larger than 0, *t*(23) = 18.49, *p* < .001, *g* = 3.69 (see, also, Table 1). The bias measure *c* was 0.30 (*SD* = 0.27), which was also significantly larger than 0, *t*(23) = 5.34, *p* < .001, *g* = 1.11, and indicated a preference for the “non-offside” response.

#### Signal-Detection Analysis–Comparison of Experiments 1–4

Further analyses compared *d’* and *c* across comparable conditions of Experiments 1–4 (i.e., Experiments 1A, 2A, 3, and 4). A one-way ANOVA with Experiment as a single between-subjects factor, and *d’* as dependent variable, revealed a significant effect, *F*(3, 96) = 10.25, *MSE* = 0.57, *p* < .001, $\eta_{partial}^{2}$ = .24. Scheffé tests showed that *d’* was significantly smaller in Experiment 4 than in each of the other experiments: All *p*s < .02, but they did not differ between the other experiments. In contrast, a one-way ANOVA with Experiment as the between-subjects factor, and *c* as dependent variable, obtained no effects, *F*(3, 96) = 1.63, *MSE* = 0.03, *p* = .19, $\eta_{partial}^{2}$ = .05.

### Discussion

Experiment 4 used photographic displays, which closely matched the spatial configuration in the more artificial displays used in Experiments 2A/B and 3, for further exploring offside judgments in laypersons. The accuracy data from Experiment 4 replicated the major findings from Experiments 2A/B and 3. Perceptual sensitivity was lower than in the previous experiments, but still significant (cf. Table 2). A positive response bias replicated the preference for the non-offside response, associated with a preponderance of non-flag errors. Moreover, the spatial separation between forward and defender had a similar effect on accuracy as in Experiments 2A/B and 3. Accuracy again decreased from level -3 to a minimum at level +1 (the first offside situation), and increased again until level +4. The figures suggest some quantitative differences in that performance was better with photos than with cliparts for non-offside situations, with the reverse being true for offside situations. As a result, the accuracy function was less symmetrical for Experiment 4 than for Experiments 2A/B and 3. Finally, the RT function was relatively flat in Experiment 4, but the fact that RTs were numerically longest (at levels 1 and 2) where accuracy was lowest indicates that the effects of spatial separation were not due to variations in speed-accuracy tradeoff, but to different levels of difficulty.

## General Discussion

Four experiments explored laypersons’ offside judgments in soccer with different types of static displays. There were four major findings. First, across experiments, we observed a consistent response bias in favor of the non-offside response in our participants. Second, in each experiment, the accuracy of judgments decreased when the spatial distance between the defender and the forward decreased. Third, when compared to conditions without feedback (i.e. Experiment 2A), providing simple feedback after reach response failed to improve performance. Finally, increasing the realism (and complexity) of the displays from across experiments decreased perceptual sensitivity (i.e. *d’*), but left the response bias unaffected. In the following, we discuss each of these results in more detail.

### Features of laypersons’ offside judgments

#### Response bias

Four experiments revealed a consistent *response bias* in offside judgments of laypersons. In particular, in our experiments, participants showed a consistent preference for the non-offside response, which led to a preponderance of non-flag errors in our experiments. When averaged across experiments, the bias index *c* was 0.20 (cf. Table 2). Notably, the bias was statistically equivalent in four different experiments that involved three types of displays (Experiments 1A, 2A, 3 and 4) and the bias was unaffected by the presentation of feedback in Experiment 3. The significant response bias in our experiments produced an asymmetry in the performance functions depicting percentage correct (PC) as a function of spatial separation (cf. Figs 3A, 4A, and 5A). In particular, the preference for the non-offside response over the offside response (in perceptually difficult situations) increased PC with small negative distances (i.e. -1 and 0) and decreased PC with small positive distances (i.e. +1). Hence, the bias measure *c* is also a summary statistic for the asymmetry in the performance function depicting PC as a function of spatial separation.

The observed bias in favor of non-offside judgments, which produces a preponderance of non-flag errors, stands in some contrast to the preponderance of flag errors that is typically found in studies of professional assistant referees [3, 4, 7, 10, 11, 12]. Yet, two obvious differences between these former studies and our study might have contributed to the different results. First, the stimulus material in previous studies typically involved movement, whereas we presented static scenes to our participants. Hence, movement-related phenomena, such as the flash-lag effect or gaze shifts, might have contributed to the preponderance of flag errors in previous studies. Second, previous studies typically tested professional assistant referees, whereas we tested laypersons. Hence, laypersons might be biased towards the non-offside response more strongly than professional assistant referees.

At a theoretical level, we distinguished between extrinsic and intrinsic sources of a response bias. Extrinsic sources include different base rates for offside and non-offside situations, or different consequences of flag and non-flag errors, respectively. However, non-offside displays (24/44) and offside displays (20/44) appeared with almost equal frequency in each block and participants were not informed about the small difference in base rates. Therefore, we do not believe that this difference affected performance. Moreover, participants were not informed about erroneous responses in our experiments (except for Experiment 3), and errors had no specific consequences in these experiments. This is probably different in real game situations, where the audience, for example, may respond differently to offside and non-offside decisions. Hence, we attribute the observed bias to an intrinsic source, although we could only speculate on its origin. Apparently, laypersons tend to decide in favor of the forward, particularly in perceptually difficult situations. Future research should address possible sources for this bias.

The observation of a robust response bias in offside judgments of laypersons has implications for the theory and practice of soccer refereeing. Regarding research, the observation of an (intrinsic) bias in laypersons underscores the potential importance of response biases in the decisions of ad-hoc referees, and probably also in those of professional referees. Previous research, however, has mostly ignored response preferences. Hence, future research should more systematically explore the sources and effects of response biases in offside-judgment tasks. Regarding practice, our results suggest that we should expect a response bias in ad hoc (assistant) referees and, therefore, should take measures to prevent too strong effects of biases on refereeing performance.

#### Spatial resolution in offside judgments

Besides investigating biases in offside judgments, we performed a more fine-grained analysis of the spatial resolution in offside judgments as previous research on the topic [3, 10]. In particular, in four experiments with different displays, we varied the spatial distance between the forward and the defender in 11 steps, symmetrically distributed around the offside line. When averaged across spatial conditions (and Experiments 2A/B—4), accuracy was about 78%, which nicely corresponds to the numbers reported in previous research on professional assistant referees [3, 7]. However, the experiments revealed a strong and consistent effect of the spatial distance between the forward and the defender on the accuracy of the offside judgments. In fact, accuracy varied between 90% and 40% across spatial distances. If we consider the smallest distances around the offside line (levels -1, 0, +1), and correct for a presumed bias effect of 15%, we observed accuracy rates around 60%, which approaches guessing rate. Across Experiments 2–4, average performance in condition -1 and condition +1 was 75% and 45%, respectively. Because the two conditions involve similar spatial separations, they are similarly difficult, and the observed difference in performance can be attributed to the effect of response bias. The bias (a preference for the non-offside response) should increase performance in non-offside conditions (i.e. condition -1) and decrease performance in offside conditions (i.e. condition +1) to a similar degree. Hence, the effect of bias on performance is approximately 30/2 = 15. If we subtract the bias effect from performance in condition -1, and add the bias effect to performance in condition +1, we get similar estimates for bias-free performance of about 60%.

Note that a preponderance of flag errors (as observed in many empirical studies) could also be explained in terms of a speed-accuracy tradeoff. The simple reason is that assistant referees have more time for processing (actual) offside situations than for processing (actual) non-offside situations because it is easier to change a non-offside decision into an offside decision than to change an offside decision into a non-offside decision. Therefore, it would be interesting to investigate whether assistant referees, who have a bias in favor of the offside response, show longer RTs for the non-offside response than for the offside response. Of course, this should be done in the laboratory because we do not have an observable response to non-offside situations in real game situations. Thus, providing the opportunity for measuring the RTs of both offside and non-offside responses is a clear advantage of laboratory studies and seems important for the understanding of underlying processes of offside judgments.

In our own data, accuracy and RT were negatively correlated, albeit weakly in Experiment 4. That is, conditions with high accuracy had short RTs and conditions with low accuracy had long RTs. In other words, we never observed a speed-accuracy tradeoff in our results. Therefore, we can conclude that the observed variations of accuracy with spatial distance reflect true variations in the difficulty of judging offside at different distances.

The fact that the accuracy of judging offside with a small distance between the forward and the defender drops well below 70% with static displays, strongly emphasizes the role of this distance variable on offside judgments. In other words, the limited spatial resolution of the visual system under typical viewing conditions (i.e. short presentation times) imposes strong limits on the ability to judge offside situations—even when explicit movement is excluded.

#### Feedback

We addressed the effects of feedback in Experiment 3 by using the same displays as in Experiment 2A (no-mask), but additionally provided simple feedback after each response (i.e. “Correct!” versus “Wrong!”). A comparison of the results from Experiments 2A and 3 revealed that feedback did not affect the participants’ bias, but had some–admittedly unexpected–effects on performance. In particular, feedback reduced the accuracy of the offside judgments (in fact, *d’* was significantly reduced), and numerically shortened RTs. Hence, the pattern of findings suggests that participants without feedback (Experiment 2A) had a stricter speed-accuracy criterion (i.e. took more time for processing the displays and reaching a decision) than did participants with feedback. The difference in speed-accuracy criteria might have, at least, two reasons. Firstly, the difference might be due to sampling error (i.e. occurred by chance). Secondly, when compared to conditions without feedback, the mostly positive feedback may have induced a more positive mood that, in turn, may have led participants to adopt a laxer speed-accuracy criterion. In sum, our results demonstrate that simple performance feedback for the duration of a single session neither reduces response biases in offside judgments of laypersons nor improves the quality of their judgments. Hence, to have these positive effects, we must either provide more training with simple feedback [27] or we must provide other types of feedback [7, 21].

### Implications of results for accounts of errors in offside judgments

The present findings are inconsistent with most previous accounts of errors in offside judgments, except for an account based on SDT [19, 20]. The *gaze-shift hypothesis* [5, 6] cannot explain our results because our task did not involve player movement and, hence, participants could not miss any movement during a gaze shift. The *flash-lag hypothesis* attributes errors in offside judgments to a mislocalization of a moving forwards’ position in the direction of movement, and hence predicts a preponderance of flag errors or false alarms [10, 15]. Because we used static displays, we would not expect a large impact of the flash-lag effect on our results, but it was still possible. Yet, in contrast to the prediction of this account, we consistently observed a preponderance of non-flag errors, or misses, in our experiments.

The *optical-error hypothesis* attributes errors in offside judgments to a mislocalization of the forwards’ relative position that results from suboptimal viewing positions of the assistant referee when standing behind or ahead of the offside line. In our experimental setup, the participants were always positioned behind (i.e. at a more central position than) the offside line. In that case, the optical-error hypothesis would predict a preponderance of flag errors (false alarms) when the forward is closer to the assistant referee than the defender (i.e., when the forward partially occludes the defender), but a preponderance of non-flag errors (misses) when the forward is farther away from the assistant referee than the defender (i.e., when the defender partially occludes the forward). Hence, the optical-error hypothesis would be able to explain the observed preponderance of non-flag errors (misses) when participants were less accurate in the latter rather than the former condition (with the two conditions being equally frequent in our experiments). A post-hoc analysis on the pooled data from Experiments 2–4 revealed the opposite result: Judgments were slightly more accurate when the defender partially occluded the forward (*M* = 85.2, *SD* = 13.2) than when the forward partially occluded the defender (*M* = 84.2, *SD* = 12.1), *F*(1, 94) = 4.85, *MSE* = 10.06, *p* < .05, $\eta_{partial}^{2}$ = .05.

A common feature of the three accounts discussed above is that they attribute errors in offside judgments to a mechanism at a perceptual stage of processing. It is conceivable, however, that errors in offside judgments may not only arise at perceptual stages, due to restrictions in perceptual sensitivity, but also at post-perceptual stages of processing, for example as a result of a response bias. SDT provides a framework for analyzing and understanding perceptual and post-perceptual effects on (offside) judgments, although the exact source of the bias observed in the present experiments has yet to be determined.

### Characteristics of displays used in present experiments

Two major findings emerged from all three types of displays (triangles, cliparts, photographs) used in our studies: the bias in favor of non-offside response and the decrease in performance with decreasing spatial distance between forward and defender. The bias in favor of the non-offside response was remarkably stable across experiments and experimental conditions. A post-hoc analysis revealed that the bias did not vary between the four experiments with unmasked displays (i.e. Experiments 1A, 2A, 3 and 4). Hence, the bias is robust and independent from the type of display used in our experiments. The latter observation is important because the fact that the bias is independent from the complexity and realism of the display provides further support for an intrinsic—rather than extrinsic—source of this bias.

The impact of the spatial separation between the forward and the defender on performance was also remarkably similar across experiments and displays (with the exception of Experiment 1B, in which performance peaked at level 0). Performance typically deteriorated when the distance between the forward and the defender decreased, revealing a V-shaped function for judgment accuracy (PC) and a bell-shaped function for reaction time (except for Experiments 1A and 1B, see below). In judgment accuracy, the performance minimum always occurred in the condition where the forward was slightly in front of the defender (i.e. at level +1), and not at level 0. Most likely, this asymmetry in the performance function is a result of the response bias that decreased accuracy at difficult offside situations (i.e. level +1) and increased accuracy at difficult non-offside situations (i.e. levels 0 and -1). In other words, with bias-free performance, the accuracy minimum would have occurred at level 0. The similar difficulty of small distances in non-offside situations (i.e. levels -1 and 0) and small distances in offside situations (i.e. level +1) is apparent in the RT functions where the longest RTs were typically observed.

The results of Experiment 1, where colored triangles represented players in the displays, differed in two notable ways from the results of the other experiments. First, judgment accuracy was highest in Experiment 1A/B, as reflected in flatter PC functions (cf. Fig 2A), indicating a simpler task than with the other displays. Second, performance in Experiment 1A/B markedly differed from performance in the remaining experiments at distance level 0. In particular, in Experiments 2A/B -4, accuracy was lower for level 0 than for level -1; in Experiment 1A/B, accuracy was equal or better for level 0 than for level -1. Similarly, in Experiments 2A/B -4, RTs were relatively long for level 0, whereas RTs were relatively short for this condition in Experiments 1A/B, producing an M-shaped RT function (cf. Fig 2B). These differences suggest that distance level 0 represented the easiest condition in Experiment 1A/B, whereas it was a very difficult condition in Experiments 2A/B -4. Presenting two colored triangles at the same horizontal location means that the object in front almost completely covers the second object, making these displays qualitatively different and, therefore, easily distinguishable from other displays. In contrast, when two clipart players are presented at the same horizontal location, there is only partial overlap between the two and, therefore, this condition is difficult to distinguish from other conditions with small distances between the players, which is more similar to natural conditions.

In summary, we recommend using displays with clipart players in future research on offside judgments because they allow for maximal experimental control over display contents and participants’ performance with clipart displays seems very similar to performance with photographic images. An additional option is to present a mask after each display. Masking increases experimental control over presentation times and decreases the risk of ceiling effects in performance. Moreover, as would be desired, masking decreased perceptual sensitivity, but it did not affect the response bias in our experiments.

## Final Remarks

Signal-detection theory provides a useful framework for investigating offside judgments in experts and laypersons. In this framework, two main variables–perceptual sensitivity and response bias–determine the quality of offside judgments. Among other variables, the limited spatial resolution of the perceptual system may constrain the perceptual sensitivity for discriminating offside from non-offside situations. In fact, our results showed that, even with static displays, offside judgments approached guessing rate for small distances between the forward and the defender. Variations in response bias provide a natural explanation for a preponderance of flag errors in some studies and a preponderance of non-flag errors in others. Our study is the first to demonstrate that even laypersons have a response bias (in favor of the offside response) when judging offside under laboratory conditions. Future studies may address the possible sources of different response biases in assistant referees. Static displays with clipart figures, as used in our experiments, may well be suited for this future work.
